# Identifying Time-Variant Predictors of Interest in Completing Brief Digital Mental Health Interventions Among Adult Survivors of Cancer: Ecological Momentary Assessment Study

**DOI:** 10.2196/69244

**Published:** 2025-12-18

**Authors:** Katharine E Daniel, James W Kinchen, Angela Chang, Patrick H Finan, Philip I Chow

**Affiliations:** 1 University of Virginia School of Medicine Department of Psychiatry and Neurobehavioral Sciences Charlottesville, VA United States; 2 University of Virginia School of Medicine Department of Publich Health Sciences Charlottesville, VA United States; 3 University of Virginia School of Medicine Department of Anesthesiology Charlottesville, VA United States

**Keywords:** digital behavioral health intervention, ecological momentary assessment, mental disorders, neoplasms, personalization

## Abstract

**Background:**

Digital microinterventions have strong potential to improve the lives of adults diagnosed with cancer. However, little is known about which types of digital microinterventions are most desired and how contextual factors may influence those preferences. This potentially limits guidance for personalized and timely digital microintervention delivery.

**Objective:**

This study aims to identify time-varying and person-level predictors of relative digital microintervention interest among adult survivors of cancer.

**Methods:**

We enrolled US adults within 5 years of a cancer diagnosis in a 5-week observational study using ecological momentary assessment. Participants (N=407) were asked 3 times a day for 5 weeks which of 9 brief, mobile-delivered interventions, if any, they would have been interested in completing within the past hour. Intervention options were (1) reducing worry, (2) reducing negative thoughts, (3) problem solving, (4) increasing positive emotions, (5) connecting with values, (6) guided relaxation, (7) getting support from others, (8) setting goals, and (9) something else. Multinomial models were used to identify demographic (ie, age), cancer-related (ie, treatment status), and psychological (ie, depression symptom severity, anxiety symptom severity, history of major depressive diagnosis, history of anxiety disorder diagnosis, and psychotherapy status) predictors of individual differences in modal intervention preference. Multilevel logistic and multilevel multinomial models were used to identify momentary negative affect, positive affect, and pain predictors of relative intervention interest.

**Results:**

Participants indicated interest in completing a digital microintervention in 87% (20,429/23,472) of completed surveys. The most frequently selected intervention option was guided relaxation (8611/20,429, 42%). Neither cancer treatment status (*χ*^2^_9_=6.5; *P*=.69) nor psychotherapy status (*χ*^2^_9_=14.0; *P*=.12) differentiated between modal intervention preferences. Participants with greater anxiety (*χ*^2^_9_=35.1; *P*<.001) and depression symptom severity (*χ*^2^_9_=23.0; *P*=.006) were less likely to modally endorse guided relaxation compared to other intervention options like increasing positive emotions, reducing negative thoughts, and getting support from others. Higher momentary negative affect and pain and lower momentary positive affect each predicted a greater likelihood to endorse interest in completing an intervention (vs not completing an intervention; *P*<.05) and to endorse interest in completing multiple interventions (vs only one; *P*<.001). Finally, higher momentary negative affect generally predicted greater interest in completing an intervention other than guided relaxation, whereas higher momentary pain generally predicted greater relative interest in guided relaxation.

**Conclusions:**

Adult survivors of cancer differ in their digital microintervention preferences between and within persons. Guided relaxation alone is less appealing to survivors of cancer when they are in greater emotional distress but may be more appealing in response to instances of increased pain.

## Introduction

### Overview

An estimated 17.9 million adult survivors of cancer were living in the United States in January 2022; by 2040, this number is projected to increase by 43.6% [1]. Navigating life after a cancer diagnosis poses significant strain on mental health and well-being [2], particularly within 5 years of diagnosis [3-6]. Across cancer types, nearly half of adult patients experience clinical or elevated levels of anxiety and nearly one-third experience clinical or elevated levels of depression [6,7]. Despite clear psychological support needs for those on and off active cancer treatment [8], adult survivors of cancer face significant barriers to accessing mental health care that include social stigma [9], financial [10] and time costs [11], and lack of available providers [12]. Promisingly, digital technologies can circumvent these barriers and increase their access to empirically supported mental health care [13,14].

Meta analyses show that both web- and mobile phone–delivered mental health interventions can effectively reduce anxiety and depression symptoms among adult survivors of cancer [15,16]. However, when using a digital intervention, adult survivors of cancer tend to prefer those that offer content specific to their changing needs and that are delivered at opportune times throughout their cancer journey [13]. For example, multiple trials show that they are less likely to engage with digital interventions that have content they perceive to be irrelevant or not useful to them [17,18] and adult survivors of cancer tend to like being able to choose which elements of an intervention system to engage with [18-20]. Mobile phone–based digital microinterventions (DMIs) [21], which are highly focused, technology-enabled interventions that can be delivered in the context of a person’s daily life with little burden on the individual, may therefore be a particularly effective approach for increasing their access to psychological support services.

There has been a dramatic increase in recent years in the number of targeted DMIs developed to address mental health concerns (eg, guided relaxation, reducing worry, social support) [22]; however, there has been relatively little work focused on developing and testing DMIs in cancer populations. Basing DMIs on cognitive behavioral therapy and acceptance and commitment therapy techniques may offer a promising starting point, because they can be readily delivered through mobile technology [23,24] and have demonstrated robust efficacy in reducing anxiety and depression symptoms in adult survivors of cancer [25,26]. Yet, for both adults with cancer and the general population, little is known about which types of DMIs people are most interested in completing throughout their daily life. Addressing this gap is critical to the effectiveness of future DMI systems given that users are most likely to engage with interventions that interest them [27]. Moreover, DMI preferences in adult survivors of cancer might differ from those in medically healthy populations given the unique stressors introduced by a cancer diagnosis. For example, survivors of cancer face high rates of fatigue and pain [28], body image dissatisfaction [29], fear of cancer recurrence [30], and disruptions to relationships [31] and occupational roles [32] throughout the cancer trajectory, all of which may impact which DMIs they are interested in completing. Further, interest among different DMIs is expected to vary within individuals across time and context [33]. Thus, if DMI systems are going to meet their promise of flexibly adapting to people’s changing psychological support needs and interests, it is important to improve prediction of time-varying user interest. Taken together, because integrating user preferences into treatment decisions is an integral component of evidence-based care [34] and interest guides engagement [27], understanding what DMIs adult survivors of cancer want and what factors shape their interest are critical to developing effective DMI systems. This study therefore sought to understand which types of DMIs (N=407) US adult survivors of cancer indicate interest in completing throughout their daily life and how contextual features influence interest endorsements.

### Aims and Hypotheses

Using data from a 5-week ecological momentary assessment (EMA) study, we aimed to identify (1) group-level interest in completing each of 9 unique DMIs, (2) person-specific features at baseline that differentiate between modally selected DMI for each survivor of cancer, and (3) importance of situation-specific features in momentary DMI interest. Specifically, we were interested in identifying situation-specific features that differentiate between when participants typically do (vs do not) express interest in completing a DMI (Aim 3a), express interest in completing a single DMI (vs multiple DMIs; Aim 3b), and express relative interest among specific DMIs (Aim 3c). Aims 1 and 2 were exploratory, whereas Aim 3 was partially hypothesis driven. We predicted that higher levels of state negative affect and state pain and lower levels of state positive affect, at both within- and between-person levels, would predict higher likelihood to endorse interest in a DMI relative to no DMI (Aim 3a) and higher likelihood to endorse interest in multiple DMIs relative to a single DMI (Aim 3b). These hypotheses are based on literature showing that individuals are more likely to sign up for help [35] and to use multiple coping strategies [36] when they are experiencing greater distress. Aim 3c was exploratory. Collectively, investigation into these aims stands to provide initial guidance on (1) which DMIs to prioritize developing based on relative user demand, (2) which DMIs a survivor of cancer is most likely to select throughout their daily life based only on information easily collected at baseline, and (3) how to consider tailoring DMI recommendations based on current user functioning. Taken together, these insights may facilitate the development of future DMIs for survivors of cancer.

## Methods

### Ethical Considerations

Study procedures were approved by the University of Virginia’s Institutional Review Board for Health Sciences Research (#230080). All participants provided informed consent prior to study enrollment. All data were deidentified prior to analysis. Participants were compensated up to US $100 for their participation, with final compensation prorated based on percentage of EMA surveys completed.

### Recruitment

Study advertisements were placed on social media outlets throughout the United States. Interested individuals were directed to complete an online preassessment screener. Individuals who were deemed pre-eligible based on their screener responses (ie, self-reported a cancer diagnosis within the past 5 years, owned a mobile phone compatible with study procedures, and were willing to download and use an app to collect study data) and who passed a background check [37] were then contacted by a study team member over email to schedule an enrollment phone call. Study team members attempted to contact individuals over email up to 3 times to schedule the phone call. During this phone call, a trained research coordinator explained study procedures and obtained informed consent. In the event of no answer to the call, a study team member attempted to reschedule the call up to 3 times. Recruitment took place between July 2023 and May 2024. A total of 1602 screening forms were submitted, of which study staff attempted to contact 1109 pre-eligible adult participants, of whom 548 were successfully contacted by study staff, and a total of 426 consented to participate.

### Participants

A total of 426 US adults within 5 years of a cancer diagnosis (stages 0-4, referred to as “survivors of cancer”) enrolled in the study. Participants were eligible regardless of mental health status, cancer treatment status, and cancer type. Although we considered narrowing our criteria to focus on a subset of adults with cancer (eg, by treatment type, survivorship status, cancer stage, or site), high levels of distress and the need for well-being support are present across virtually all subgroups. Given the limited research on DMI preferences in adults with cancer, we decided to keep our scope as broad as possible. The cancer types included in the resulting dataset represent some of the most common forms in the United States. Of the 426 who were enrolled, 19 were excluded (n=10 did not initiate the EMA and n=9 requested to withdraw), resulting in a final sample of 407. Sample demographics are reported in Table 1.

**Table 1:** Sample demographics.

Participant characteristics				Frequency
Age (years), mean (SD)				48.73 (12.23)
**Sex, n (%)**				
	Male			37 (9.1)
	Female			367 (90.2)
	Some other sex			3 (0.7)
**Race, n (%)**				
	White			351 (87)
	Black			20 (4.9)
	American Indian/Alaska Native			6 (1.5)
	Asian			17 (4.2)
	Native Hawaiian/Pacific Islander			1 (0.3)
	Some other race			10 (2.5)
**Ethnicity, n (%)**				
	Non-Hispanic			371 (93)
	Hispanic			30 (7.5)
**Cancer information, n (%)**				
	**Type of cancer**			
		Breast	232 (57.3)	
		Prostate	16 (4.0)	
		Lung	13 (3.2)	
		Colorectal	21 (5.2)	
		Endometrial, ovarian, or cervical	33 (8.2)	
		Non-Hodgkin lymphoma	12 (3.0)	
		Some other cancer^a^	78 (19.3)	
	In active cancer treatment			84 (21.9)
**Psychiatric information**				
	In active therapy, n (%)			133 (33)
	History of major depressive disorder, n (%)			198 (49)
	History of anxiety disorder, n (%)			214 (53)
	PHQ-8^b^, mean (SD)			7.76 (5.25)
	GAD-7^c^, mean (SD)			5.64 (4.95)

^a^Includes melanoma, bladder, kidney, pancreas, leukemia, thyroid, Hodgkin lymphoma, and brain cancers.

^b^PHQ-8: Patient Health Questionnaire-8.

^c^GAD-7: Generalized Anxiety Disorder-7.

### Procedure

Participants enrolled to participate in a 5-week EMA study. Prior to starting the EMA phase, participants completed a battery of self-report questionnaires through REDCap (Research Electronic Data Capture; Vanderbilt University) [38] measuring demographics, cancer diagnosis and treatment history, and psychological functioning. In the EMA phase, participants received 3 surveys on their mobile phones each day through the Effortless Assessment Research System (EARS) mobile app [39]. The EARS mobile app has been successfully used in a range of populations [40-42], and EMA is being increasingly used within the cancer context [43]. One survey was delivered randomly within each of the following time blocks: 8 AM to 10 AM, 1 PM to 3 PM, and 7 PM to 9 PM. Participants had up to 2 hours to complete each survey.

### Measures

#### Trait Measures

At baseline, participants reported their age, gender, race, cancer diagnosis, whether they were in active cancer treatment (yes vs no), whether they were currently in psychotherapy for anxiety or depression symptoms (yes vs no), whether they had ever been diagnosed with major depressive disorder (yes vs no), and whether they had ever been diagnosed with an anxiety disorder (yes vs no). Participants also completed the Generalized Anxiety Disorder-7 (GAD-7) [44] and Patient Health Questionnaire-8 (PHQ-8) [45] to measure generalized anxiety and depressive symptom severity, respectively, over the previous 2 weeks. Higher GAD-7 and PHQ-8 scores indicate greater generalized anxiety and depression symptom severity, respectively. Cronbach α for GAD-7 and PHQ-8 was 0.91 and 0.87, respectively.

#### EMA Measures

To measure interest in completing different interventions, participants were asked at each EMA survey to select which type of exercise(s) they would have been interested in completing, had the exercises been offered as a “brief guided exercise on your phone sometime in the last hour,” using a check-all-that-apply list. Eight interventions drawn from cognitive behavioral therapy and acceptance and commitment therapy were included, along with a ninth option of “other (something else).” The interventions listed were (1) reducing worry, (2) reducing negative thoughts, (3) problem solving, (4) increasing positive emotions, (5) connecting with values, (6) guided relaxation, (7) getting support from others, and (8) setting goals. These intervention names were intended to be self-explanatory and descriptive of potential user needs. If participants selected “other (something else),” they had the option to write in additional intervention options. Participants could progress through the survey without selecting any of the available options, thereby indicating no momentary interest in completing an intervention.

To measure state affect, participants were asked to rate the degree to which they felt happy, cheerful, pleased, sad, afraid, and miserable over the past hour using a 0 (not at all) to 10 (very much) scale for each emotion word. To increase internal validity, positive affect is the sum of the first 3 emotion words. Negative affect is the sum of the last 3 emotion words, with higher values indicating greater levels of momentary positive and negative affect, respectively. Negative and positive affect ranged from 0 to 30, with higher values indicating more intense affect. Participants were also asked to rate their pain over the past hour using a 0 (no pain) to 10 (worst pain imaginable) scale.

### EMA Data Processing Steps

Only surveys that participants responded to were logged. We removed logged EMA surveys where all items were missing (n=2 out of 23,474; <0.008%). Surveys in which the participant did not select any of the available interventions from the response options (including other) but responded to other items in the survey were recoded to capture no interest in any intervention option.

#### Identifying the Most Preferred Intervention at the Person Level (Outcome for Aim 2)

We counted the number of times that each intervention was selected by a given participant across the full study. For each participant, the intervention with the highest count was considered their most preferred intervention overall. If multiple interventions were equally most frequently endorsed by a given participant, the most preferred intervention was coded as “multiple interventions.” Because we were interested in identifying participants’ most preferred intervention, we only used surveys where at least 1 intervention was selected.

#### Identifying Intervention Interest at the EMA Survey Level (Outcome for Aim 3c)

To capture which type(s) of interventions were of most interest to participants at the EMA survey level, all surveys with 1 selected intervention were coded according to the selected intervention. Surveys with more than 1 selected intervention were coded as “multiple,” regardless of how many and which interventions were selected. We treated “multiple” surveys in this way for analytic parsimony.

### Plans for Analysis

All analyses were conducted in R (version 4.3.3) [46].

To identify person-level predictors that explain individual differences in participants' most preferred interventions (Aim 2), we ran a series of multinomial logistic regressions using the *nnet* package [47]. Specifically, a multinomial logistic regression was conducted to examine the effect of each predictor (eg, age) in isolation on the log odds that a participant preferred each of the interventions relative to guided relaxation. Guided relaxation was used as the reference group in all models because it was the most frequently selected intervention across participants. Results should be interpreted as the difference in the likelihood of an individual’s most preferred intervention relative to guided relaxation for each 1-unit increase in the predictor.

To identify situation-level characteristics that predict likelihood to endorse interest in any intervention versus no interest (Aim 3a), we ran a series of multilevel logistic regressions using the *lme4* package [48]. The outcome variable was coded 0 if at least 1 intervention was endorsed at that survey and 1 if no intervention was endorsed. Each model included a random intercept for participant, and each fixed effect of interest (negative affect, positive affect, or pain) was decomposed into its between- and within-person components. The between-person term represents the average level of affect or pain reported across the study by that participant, which was grand mean centered. The within-person term, by contrast, represents the level of affect or pain reported by the participant at each survey, which was person mean centered to capture deviations from participant’s personal average. All models controlled for whether the survey was delivered in the first, second, or third EMA survey block.

To identify situation-level predictors of likelihood to express interest in one versus multiple interventions (Aim 3b), we removed all surveys in which no intervention was endorsed. Then, using an identical modeling structure to Aim 3a, we ran a series of multilevel logistic regressions predicting the updated outcome. The outcome variable was coded 0 if only 1 intervention was endorsed, regardless of which one, and 1 if more than 1 intervention was endorsed.

To identify situation-level predictors that explain differences in relative likelihood to endorse each of the interventions at any given survey (Aim 3c), we ran a series of multilevel multinomial logistic regressions using the *mclogit* package [49]. Only surveys in which a single intervention was endorsed were included. As in Aim 2, we set guided relaxation as the reference group in all models. Following the multilevel modeling structure used in Aims 3a and 3b, we included a random intercept for participant, decomposed each fixed effect of interest (negative affect, positive affect, or pain) into its between- and within-person components, and controlled for whether the survey was delivered in the first, second, or third EMA survey block.

### Power analyses

Sample size was determined according to parameters needed for the primary aims of the parent data collection, which was to examine change in affect in survivors of cancer. Following established guidelines for reporting sample size justifications based on resource constraints [50], we conducted a power analysis of predetermined effect sizes via simulation. The estimated minimally detectable effect size for these analyses, assuming 80% power, was odds ratio (OR) of 1.07, whereas an OR of 2 is considered a small effect size within clinical research [51]. Therefore, given our sample and model structure, we are adequately powered to detect even small effect sizes for determining person-level predictors that explain individual differences in participants’ most preferred interventions.

### Transparency and Openness

Analysis scripts are openly provided on the Open Science Framework [52]. Deidentifiable data are available upon reasonable request. Given the exploratory aims of this work, we follow guidance not to correct for multiple comparisons [53].

## Results

### Overview

A total of 23,472 EMA surveys were completed across the 407 participants (overall EMA response rate, 55%). We provide detailed descriptive information to show which interventions tended to be selected alone and in combination in Table 2. No intervention was selected in 13% (3043/23,472) of all completed surveys. Therefore, adult survivors of cancer selected at least 1 intervention 87% (20,429/23,472) of the time. This suggests that, when able to respond to a survey, survivors of cancer were much more often interested in receiving some form of support through their phone than not.

**Table 2:** Frequency of brief intervention selections.

Selected brief intervention			Frequency^a^, n (%)
No brief intervention selected			3043 (13)
**Brief intervention selected alone**			9528 (41)
	Guided relaxation	3221 (14)	
	Increasing positive emotions	1474 (6)	
	Reducing worry	1200 (5)	
	Setting goals	860 (4)	
	Reducing negative thoughts	690 (3)	
	Problem solving	501 (2)	
	Connecting with values	489 (2)	
	Getting support from others	364 (2)	
	Other	729 (3)	
**Brief interventions selected in combination**			10,901 (46)
	Reducing worry and reducing negative thoughts	616 (3)	
	Reducing worry, reducing negative thoughts, and increasing positive emotions	575 (2)	
	All brief interventions (not including “other”)	480 (2)	
	Increasing positive emotions and guided relaxation	430 (2)	
	Reducing worry, reducing negative thoughts, increasing positive emotions, and guided relaxation	325 (1)	
	reducing worry and guided relaxation	304 (1)	
	Guided relaxation and setting goals	290 (1)	
	Reducing negative thoughts and increasing positive emotions	261 (1)	

^a^A total of 2^9^ (ie, 512) intervention combinations were possible. We observed 322 unique combinations. To make the table legible, we report only frequencies for combinations observed >1% of the time across all observations.

### Aim 1: Intervention Selection Frequency

As presented in Table 2, the most often selected intervention across the sample was guided relaxation (8611/20,429, 42%), followed, in order, by increasing positive emotions (7881/20,429, 39%), reducing worry (7162/20,429, 35%), reducing negative thoughts (6535/20,429, 32%), problem solving (4148/20,429, 20%), setting goals (4081/20,429, 20%), getting support from others (3661/20,429, 18%), connecting with values (3534/20,429, 17%), and other (1007/20,429, 5%). When investigating the write-in responses for “other,” recommendations included encouragement to engage in physical exercise (eg, yoga, stretching, walking), help finding opportunities to share joy and peace with others, help with sleep, gratitude practice, and help managing physical symptoms (eg, brain fog, pain management). Percentages are reported with respect to the total number of surveys where at least 1 intervention was selected (n=20,429). Counts sum to more than the total number of completed EMA surveys because more than 1 intervention could be selected at the same time.

Although guided relaxation was the most frequently selected intervention overall, 69% (281/407) of participants selected some other option as their most preferred intervention, suggesting individual variability across survivors of cancer regarding their most frequently desired intervention.

### Aim Two: Person-Level Predictors of Overall Intervention Preference

#### Results From Aim 2 Multinomial Models

Results from all multinomial logistic models with significant predictors are reported in Table 3.

**Table 3:** Output from multinomial models.

Outcome			Coefficient					95% CI					SE					Wald statistic^a^				Odds ratio, 95% CI
			Intercept		Estimate		Intercept			Estimate		Intercept			Estimate		Intercept		Estimate			
**Age as predictor**																						
	Increasing positive emotions	–0.95		0.009		–2.15 to 0.26			–0.02 to 0.03		0.62			0.012		–1.54			0.714	1.01 (0.98 to 1.03)		
	Reducing worry	–1.45		0.015		–2.76 to -.14			–0.01 to 0.04		0.67			0.013		–2.17			1.114	1.01 (0.99 to 1.04)		
	Multiple	–1.13		0.006		–2.47 to .21			–.02 to .03		0.69			0.014		–1.65			0.405	1.01 (0.98 to 1.03)		
	Reducing negative thoughts	–1.72		0.003		–3.47 to –0.03			–0.03 to 0.04		0.89			0.018		–1.93			0.192	1.00 (0.97 to 1.04)		
	Setting goals	–1.50		–0.009		–3.50 to 0.50			–0.05 to 0.03		1.02			0.021		–1.47			–0.421	0.99 (0.95 to 1.03)		
	Other	–4.60		0.049^b^		–7.14 to –2.05			0.003 to 0.10		1.30			0.024		–3.54			2.067	1.05 (1.003 to 1.10)		
	Getting support from others	–4.05		0.040		–6.45 to –1.66			–0.004 to 0.08		1.22			0.023		–3.32			1.767	1.04 (0.996 to 1.09)		
	Connecting with values	–0.10		–0.047		–2.29 to 2.10			–0.10 to 0.004		1.12			0.026		–0.09			–1.811	0.95 (0.91 to 1.004)		
	Problem solving	–9.42		0.120^c^		–14.23 to -4.61			0.04 to 0.20		2.45			0.039		–3.84			3.060	1.13 (1.04 to 1.22)		
**Major depressive disorder diagnosis as predictor**																						
	Increasing positive emotions	–0.80		0.568		–1.20 to –0.40			–0.02 to 1.15		0.20			0.298		–3.94			1.908	1.76 (0.98 to 3.16)		
	Reducing worry	–0.92		0.384		–1.34 to –0.51			–0.24 to 1.01		0.21			0.319		–4.35			1.204	1.47 (0.79 to 2.74)		
	Multiple	–1.14		0.563		–1.59 to –0.69			–0.09 to 1.21		0.23			0.333		–4.95			1.691	1.76 (0.91 to 3.37)		
	Reducing negative thoughts	–2.28		1.296^c^		–3.00 to –1.55			0.39 to 2.20		0.37			0.463		–6.13			2.801	3.66 (1.48 to 9.06)		
	Setting goals	–2.28		0.709		–3.00 to –1.55			–0.29 to 1.71		0.37			0.509		–6.13			1.394	2.03 (0.75 to 5.50)		
	Other	–2.57		0.891		–3.40 to –1.73			–0.20 to 1.99		0.42			0.558		–6.05			1.597	2.44 (0.82 to 7.28)		
	Getting support from others	–2.97		1.584^c^		–3.97 to –1.97			0.40 to 2.77		0.51			0.606		–5.79			2.615	4.87 (1.49 to 15.98)		
	Connecting with values	–2.16		–0.102		–2.85 to –1.47			–1.25 to 1.05		0.35			0.587		–6.13			–0.174	0.90 (0.29 to 2.85)		
	Problem solving	–2.75		–0.025		–3.65 to –1.84			–1.50 to 1.45		0.46			0.753		–5.96			–0.033	0.98 (0.22 to 4.27)		
**Depression symptom severity as predictor**																						
	Increasing positive emotions	–1.05		0.070^b^		–1.57 to –0.52			0.01 to 0.13		0.27			0.030		–3.88			2.326	1.07 (1.01 to 1.14)		
	Reducing worry	–1.21		0.062		–1.77 to –0.64			–0.0003 to 0.13		0.29			0.032		–4.19			1.969	1.06 (1.0003 to 1.13)		
	Multiple	–1.70		0.098^c^		–2.33 to –1.07			0.03 to 0.16		0.32			0.033		–5.29			2.979	1.10 (1.03 to 1.18)		
	Reducing negative thoughts	–2.43		0.111^c^		–3.25 to –1.61			0.04 to 0.19		0.42			0.040		–5.82			2.747	1.12 (1.03 to 1.21)		
	Setting goals	–1.48		–0.076		–2.29 to –0.67			–0.20 to 0.05		0.42			0.063		–3.57			–1.219	0.93 (0.82 to 1.05)		
	Other	–2.44		0.048		–3.41 to –1.48			–0.06 to 0.15		0.49			0.054		–4.97			0.882	1.05 (0.94 to 1.17)		
	Getting support from others	–3.26		0.139^c^		–4.35 to –2.17			0.04 to 0.24		0.56			0.049		–5.85			2.811	1.15 (1.04 to 1.27)		
	Connecting with values	–2.61		0.060		–3.62 to –1.60			–.05 to .17		0.51			0.055		–5.07			1.098	1.06 (0.95 to 1.18)		
	Problem solving	–2.95		0.031		–4.21 to –1.68			–0.11 to 0.18		0.65			0.074		-4.56			0.423	1.03 (0.89 to 1.19)		
**Generalized anxiety symptom severity as predictor**																						
	Increasing positive emotions	–1.07		0.102^c^		–1.52 to –0.61			0.03 to 0.17		0.23			0.035		–4.58			2.889	1.11 (1.03 to 1.19)		
	Reducing worry	–1.60		0.157^d^		–2.11 to –1.09			0.09 to 0.23		0.26			0.035		–6.13			4.424	1.17 (1.09 to 1.25)		
	Multiple	–1.61		0.140^d^		–2.14 to –1.09			0.07 to 0.21		0.27			0.037		–6.02			3.810	1.15 (1.07 to 1.24)		
	Reducing negative Thoughts	–2.27		0.133^c^		–2.96 to –1.58			0.04 to 0.22		0.35			0.046		–6.47			2.897	1.14 (1.04 to 1.25)		
	Setting goals	–1.89		–0.005		–2.62 to –1.16			–0.14 to 0.13		0.37			0.069		–5.09			–0.069	1.00 (0.87 to 1.14)		
	Other	–2.37		0.061		–3.18 to –1.56			–0.06 to 0.19		0.41			0.064		–5.72			0.956	1.06 (0.94 to 1.21)		
	Getting support from others	–3.17		0.185^d^		–4.09 to –2.25			0.08 to 0.29		0.47			0.051		–6.75			3.596	1.20 (1.09 to 1.33)		
	Connecting with values	–2.67		0.104		–3.53 to –1.81			–0.01 to 0.22		0.44			0.060		–6.07			1.737	1.11 (0.99 to 1.25)		
	Problem solving	–2.72		–0.001		–3.78 to –1.67			–0.19 to 0.19		0.54			0.099		–5.05			–0.006	1.00 (0.82 to 1.21)		

^a^Wald statistics ≥|2| are statistically significant at the .05 level.

^b^*P*<.05.

^c^*P*<.01.

^d^*P*<.001.

#### Age

Specifying age provided unique predictive information relative to the null model (*χ*^2^_9_=24.5; *P*=.004). Specifically, as participant age increased, they became increasingly likely to prefer problem solving and “other” relative to guided relaxation alone. No other coefficients were statistically significant.

#### Cancer Treatment Status

Specifying cancer treatment status provided no unique predictive information relative to the null model (*χ*^2^_9_=6.5; *P*=.69).

#### Psychotherapy Status

Specifying psychotherapy treatment status provided no unique predictive information relative to the null model (*χ*^2^_9_=14.0; *P*=.12).

#### History of Major Depressive Disorder

Specifying depression diagnostic history provided unique predictive information relative to the null model (*χ*^2^_9_=17.9; *P*=.04). Specifically, results showed that participants with (vs without) a prior depression diagnosis were more likely to prefer reducing negative thoughts and getting support from others relative to guided relaxation alone. No other coefficients were statistically significant.

#### Current Depression Symptom Severity

Specifying current depression symptom severity provided unique predictive information relative to the null model (*χ*^2^_9_=23.0; *P*=.006). Specifically, as depression symptom severity increased, participants became increasingly likely to prefer increasing positive emotions, multiple interventions (which could include guided relaxation), reducing negative thoughts, and getting support from others relative to guided relaxation alone.

#### History of an Anxiety Disorder

Specifying anxiety diagnostic history provided no unique predictive information relative to the null model (*χ*^2^_9_= 8.5; *P*=.49).

#### Current Generalized Anxiety Symptom Severity

Specifying current generalized anxiety symptom severity provided unique predictive information relative to the null model (*χ*^2^_9_=35.1; *P*<.001). Specifically, as generalized anxiety symptom severity increased, participants became increasingly likely to prefer increasing positive emotions, reducing worry, multiple interventions (which could include guided relaxation), reducing negative thoughts, and getting support from others relative to guided relaxation alone.

### Aim 3: Situation-Level Predictors of Intervention Interest

#### Aim 3a: Likelihood to Express Interest in Any Intervention Versus No Interest

EMA survey block was not significant in any Aim 3a model.

##### Negative Affect

Higher levels of within-person negative affect (b=–0.07; SE=0.01; *P*<.001; OR 0.93, 95% CI 0.91-0.95) and between-person negative affect (b=–0.13; SE=0.05; *P*=.01; OR 0.88, 95% CI 0.79-0.97) were associated with a greater likelihood of reporting interest in an intervention. Mean negative affect when interest in an intervention was endorsed was 3.99 (SD 5.60) and 2.67 (SD 4.39) when no interest in any intervention was endorsed.

##### Positive Affect

Higher levels of within-person positive affect were associated with a lower likelihood of reporting interest in an intervention (b=0.03; SE=0.01; *P*<.001; OR 1.03, 95% CI 1.02-1.04). Between-person positive affect was not significant (b=–0.002; SE=0.03; *P*=.95; OR 0.98, 95% CI 0.982-0.983). Mean positive affect when interest in an intervention was endorsed was 17.1 (SD 8.02) and 17.9 (SD 8.79) when no interest in any intervention was endorsed.

##### Pain

Higher levels of within-person pain (b=–0.05; SE=0.03; *P*=.03; OR 0.95, 95% CI 0.90-1.00) and between-person pain (b=–0.32; SE=0.11; *P*=.002; OR 0.72, 95% CI 0.59-0.89) were associated with a greater likelihood of reporting interest in an intervention. Mean pain when interest in an intervention was endorsed was 2.22 (SD 2.31) and 1.69 (SD 2.02) when no interest in any intervention was endorsed.

##### Lagged Effects

Re-running the above 3 models with the within-person predictor lagged revealed that if a survivor of cancer’s current negative affect is relatively high compared to their personal average, they are more likely to report interest in an intervention at the next time point than if their current negative affect is relatively low (b=–0.03; SE=0.01; *P*<.001; OR 0.97, 95% CI 0.95-0.99). Neither the lagged effects of within-person positive affect (b=0.002; SE=0.01; *P*=.74; OR 1, 95% CI 0.99-1.01) nor within-person pain (b=–0.01; SE=0.02; *P*=.74; OR 0.99, 95% CI 0.95-1.04) were significant.

#### Aim 3b: Likelihood to Express Interest in Any Single Intervention Versus Multiple Interventions

EMA survey block was not significant in any Aim 3b model.

##### Negative Affect

Higher levels of within-person negative affect (b=0.08; SE=0.01; *P*<.001; OR 1.09, 95% CI 1.05-1.82) and between-person negative affect (b=0.24; SE=0.03; *P*<.001; OR 1.27, 95% CI 1.19-1.35) were associated with a greater likelihood of reporting interest in multiple interventions. Mean negative affect on EMAs when 1 intervention was endorsed was 2.75 (SD 4.52) and 5.06 (SD 6.20) when multiple interventions were endorsed.

##### Positive Affect

Higher levels of within-person positive affect (b=–0.05; SE=0.004; *P*<.001; OR 0.95, 95% CI 0.95-0.96) and between-person positive affect (b=–0.12; SE=0.02; *P*<.001; OR 0.89, 95% CI 0.85-0.92) were associated with a lower likelihood of reporting interest in multiple interventions. Mean positive affect on EMAs when 1 intervention was endorsed was 18.5 (SD 8.03) and 15.9 (SD 7.80) when multiple interventions were endorsed.

##### Pain

Higher levels of within-person pain (b=0.07; SE=0.02; *P*<.001; OR 1.07, 95% CI 1.03-1.11) and between-person pain (b=0.37; SE=0.07; *P*<.001; OR 1.45, 95% CI 1.27-1.66) were associated with a greater likelihood of reporting interest in multiple interventions. Mean pain on EMAs when 1 intervention was endorsed was 1.84 (SD 2.22) and 2.55 (SD 2.34) when multiple interventions were endorsed.

##### Lagged Effects

Re-running the above 3 models with the within-person predictor lagged uncovered that if a participant’s current negative affect is relatively high, they are more likely to report interest in multiple interventions at the next time point than if their current negative affect was relatively low (b=0.03; SE=0.01; *P*<.001; OR 1.03, 95% CI 1.02-1.04). Moreover, if a participant’s current positive affect is relatively high, they are less likely to report interest in multiple interventions at the next time point than if their current positive affect was relatively low (b=–0.02; SE=0.004; *P*<.001; OR 0.99, 95% CI 0.98-0.99). The lagged effect of within-person pain was not significant (b=0.03; SE=0.02; *P*=.15; OR 1.03, 95% CI 0.99-1.06).

#### Aim 3c: Likelihood to Express Relative Interest Among Specific Interventions

##### Negative Affect

Higher levels of within- and between-person negative affect were associated with a significantly greater likelihood of reporting interest in each of the following interventions relative to guided relaxation: increasing positive emotions (within: b=0.05; SE=0.01; *P*<.001 and between: b=0.20; SE=0.03; *P*<.001), reducing worry (within: b=0.12; SE=0.01; *P*<.001 and between: b=0.25; SE=0.04; *P*<.001), reducing negative thoughts (within: b=0.15; SE=0.01; *P*<.001 and between: b=0.23; SE=0.04; *P*<.001), problem solving (within: b=0.10; SE=0.02; *P*<.001 and between: b=0.14; SE=0.04; *P*<.001), getting support from others (within: b=0.15; SE=0.02; *P*<.001 and between: b=0.22; SE=0.04; *P*<.001), and another intervention (within: b=0.10; SE=0.02; *P*<.001 and between: b=0.14; SE=0.04; *P*<.001). Lower levels of within-person negative affect (but not between-person negative affect) were associated with a significantly greater likelihood of reporting interest in setting goals relative to guided relaxation (b=–0.06; SE=0.02; *P*=.009). Higher levels of between-person negative affect (but not within-person negative affect) were associated with a significantly greater likelihood of reporting interest in connecting with values relative to guided relaxation (b=0.13; SE=0.04; *P*<.001).

##### Positive Affect

Higher levels of within- and between-person positive affect were associated with a significantly lower likelihood of reporting interest in each of the following interventions relative to guided relaxation: increasing positive emotions (within: b=–0.03; SE=0.01; *P*<.001 and between: b=–0.08; SE=0.02; *P*<.001), reducing worry (within: b=–0.05; SE=0.01; *P*<.001 and between: b=–0.09; SE=0.02; *P*<.001), reducing negative thoughts (within: b=–0.09; SE=0.01; *P*<.001 and between: b=–0.08; SE=0.02; *P*<.001), problem solving (within: b=–0.05; SE=0.01; *P*<.001 and between: b=–0.05; SE=0.02; *P*=.01), getting support from others (within: b=–0.05; SE=0.01; *P*< .001 and between: b=–0.10; SE=0.02; *P*<.001), and another intervention (within: b=–0.03; SE=0.01; *P*=.01 and between: b=–0.05; SE=0.02; *P*=.03). Higher levels of within-person positive affect (but not between-person positive affect) were associated with a significantly greater likelihood of reporting interest in setting goals (b=0.06; SE=0.01; *P*<.001) and connecting with values (b=0.05; SE=0.01; *P*<.001) relative to guided relaxation.

##### Pain

Higher levels of within-person pain and lower levels of between-person pain were associated with a significantly lower likelihood of reporting interest in increasing positive emotions (within: b=–0.16; SE=0.03; *P*<.001 and between: b=0.19; SE=0.06; *P*<.01) relative to guided relaxation. Higher levels of within-person pain (but not between-person pain) were associated with lower likelihood of reporting interest in problem solving (b=–0.13; SE=0.05; *P*=.02), setting goals (b=–0.22; SE=0.05; *P*<.001), and connecting with values (b=–0.17; SE=0.05; *P*=.002) relative to guided relaxation. Higher levels of between-person pain (but not within-person pain) were associated with greater likelihood of reporting interest in reducing negative thoughts (b=0.16; SE=0.07; *P*=.02), reducing worry (b=0.28; SE=0.07; *P*<.001), getting support from others (b=0.27, SE=0.07; *P*<.001), and another intervention (b=0.23; SE=0.07, *P*=.001) relative to guided relaxation.

##### Lagged Effects

Re-running the above 3 models with the within-person predictor lagged uncovered a few significant lagged effects for negative and positive affect. Specifically, if a survivor of cancer’s current negative affect is relatively high, they are more likely to report interest in getting support from others (b=0.05; SE=0.02; *P*=.006) and another intervention (b=0.04; SE=0.02; *P*=.006) relative to guided relaxation at the next time point than if their current negative affect was relatively low. If a survivor of cancer’s current positive affect is relatively high, they are more likely to report interest in setting goals relative to guided relaxation (b=0.03; SE=0.01; *P*=.003) and less likely to report interest in reducing negative thoughts (b=–0.03; SE=0.01; *P*=.003) and increasing positive emotions (b=–0.02; SE=0.01; *P*=.01) relative to guided relaxation at the next time point than if their current negative affect was relatively low. No significant lagged effects for pain were observed.

## Discussion

### Overview

This study is the first to investigate personal and situational predictors of interest in specific types of DMIs among adults diagnosed with cancer. Participants reported interest in completing a DMI in the vast (nearly 90%) majority of cases they were available to complete a survey. Moreover, while guided relaxation was the most popular intervention option selected overall, there were important personal and contextual factors that were associated with the DMIs survivors of cancer most desired. Therefore, findings from this study not only highlight that adult survivors of cancer are frequently interested in being administered DMIs throughout their daily lives, but also underscore some potentially important considerations for tailoring intervention content to the individual and circumstance in question. A conceptual overview of all statistically significant results is provided in Table 4.

**Table 4:** Conceptual overview of model-based study findings.

Aim and predictor		Finding
**Aim 2: Identify person-level predictors that explain individual differences in their most preferred interventions**		
	Age	Older age was associated with a significantly greater likelihood to prefer problem solving and some other intervention relative to guided relaxation.
	Cancer treatment status	No significant results.
	Psychotherapy status	No significant results.
	Depression symptom severity	Greater depression symptom severity was associated with a significantly greater likelihood to prefer increasing positive emotions, reducing negative thoughts, getting support from others, and multiple interventions relative to guided relaxation.
	Anxiety symptom severity	Greater anxiety symptom severity was associated with a significantly greater likelihood to prefer increasing positive emotions, reducing negative thoughts, reducing worry, getting support from others, and multiple interventions relative to guided relaxation.
	Major depression diagnosis	Being diagnosed with major depressive disorder was associated with a significantly greater likelihood to prefer reducing negative thoughts and getting support from others relative to guided relaxation.
	Anxiety disorder diagnosis	No significant results.
**Aim 3a: Identify situation-specific features that differentiate between when participants do (vs do not) express interest in completing a DMI^a^.**		
	Negative affect	Higher within- and between-person negative affect was associated with a greater likelihood of being interested in an intervention.
	Positive affect	Higher within-person positive affect was associated with a lower likelihood of being interested in an intervention.
	Pain	Higher within- and between-person pain was associated with a greater likelihood of being interested in an intervention.
**Aim 3b: Identify situation-specific features that differentiate between when participants express interest in completing a single DMI (vs multiple DMIs).**		
	Negative affect	Higher within- and between-person negative affect was associated with a greater likelihood of being interested in multiple interventions.
	Positive affect	Higher within- and between-person positive affect was associated with a lower likelihood of being interested in multiple interventions.
	Pain	Higher within- and between-person pain was associated with a greater likelihood of being interested in multiple interventions.
**Aim 3c: Identify situation-specific features that explain momentary differences in relative interest among specific DMIs. For parsimony, we only focus on within-person effects in this table.**		
	Negative affect	Momentary spikes in negative affect were associated with an increased likelihood to select increasing positive emotions, reducing worry, reducing negative thoughts, problem solving, getting support from others, and some other intervention relative to guided relaxation. Momentary spikes in negative affect were associated with a decreased likelihood to select setting goals relative to guided relaxation.
	Positive affect	Momentary spikes in positive affect were associated with a decreased likelihood to select increasing positive emotions, reducing worry, reducing negative thoughts, problem solving, getting support from others, and some other intervention relative to guided relaxation. Momentary spikes in positive affect were associated with an increased likelihood to select setting goals relative to guided relaxation.
	Pain	Momentary spikes in pain were associated with a decreased likelihood to select increasing positive emotions, problem solving, setting goals, and connecting with values relative to guided relaxation.

^a^DMI: digital microintervention.

Findings from this study support the value of personalizing intervention recommendations to the individual [13]. We found evidence that as age increased, participants were more likely to primarily endorse problem-solving relative to guided relaxation, and participants with (vs without) a history of major depressive disorder were more likely to prefer reducing negative thoughts and getting support from others relative to guided relaxation. Older adults in our sample might have gravitated toward problem solving support due to age-related changes in problem solving ability [54,55], whereas participants with a history of depression may have been motivated to target core depression symptoms of repetitive negative thoughts [56] and social isolation [57]. Similarly, we found that greater current depression and anxiety symptom severity were associated with a greater likelihood of generally endorsing interest in interventions that increase positive emotions, reduce negative thoughts, offer support from others, or multiple interventions (which could include guided relaxation), relative to guided relaxation alone. Higher current anxiety symptom severity was additionally associated with a greater likelihood to primarily endorse reducing worry relative to guided relaxation, which is expected given the prominent role of uncontrollable worry in anxiety [58]. Collectively, these findings suggest adult survivors of cancer without a past or current history of depression and anxiety may most desire DMIs such as guided relaxation that offer immediate benefit, while older survivors and those with a past and current history of depression and anxiety may prefer multiple DMI recommendations weighted toward skills acquisition and increasing positive affect that target specific aspects of their mental functioning. Notably, whether participants were in active cancer treatment did not impact their relative intervention preferences, which underscores the importance of offering accessible mental health care to adult survivors of cancer in need regardless of their cancer stage or treatment status [59].

This study demonstrates that DMI preference is not static for adult survivors of cancer, which is consistent with ongoing efforts to dynamically tailor interventions to users across circumstances [60,61]. When participants endorsed an intervention option, spikes in their negative affect predicted relatively less interest in guided relaxation compared to problem solving and increasing positive emotions, among other DMI options. Conversely, spikes in pain predicted relatively greater interest in guided relaxation relative to problem solving, increasing positive emotions, setting goals, and connecting with values. These findings indicate that momentary assessments of physical and emotional states may be useful when identifying relative momentary DMI interest. Notably, higher between-person pain was associated with relatively lower interest in guided relaxation relative to increasing positive emotions, reducing negative thoughts, getting support from others, and reducing worry. Taken together, these findings indicate that adult survivors of cancer with chronically elevated pain may be less interested in guided relaxation relative to DMIs that directly tackle thoughts and emotions associated with their pain, whereas those with fluctuating pain may be more interested in relaxation-focused DMIs to mitigate pain in the moment.

Findings from this study are consistent with previous work showing that poorer psychological functioning predicts greater interest in digital interventions [27,35]. Specifically, participants were more likely to endorse interest in completing a DMI (vs no interest) during moments of relatively higher within- and between-person negative affect and pain, and lower within-person positive affect. Moreover, we found that relative spikes in negative affect and pain (but not positive affect) predicted greater likelihood of intervention interest at the next moment (average time between surveys is 13.49 hours, SD 18.00). These differential lagged effects are in line with the finding that negative emotions tend to linger longer than positive emotions [62]. As such, DMIs aiming to minimize user burden may benefit from focusing on momentary assessments of negative affect to guide the timing of future DMI deliveries.

Our findings advance the literature aiming to identify which DMI to offer at the opportune moment [33]. Rather than trying to identify which single DMI to offer, researchers may wish to offer multiple DMIs to personalize their interventions. In the instances in which they stated a preference for a DMI, adult survivors of cancer in the present sample were interested in more than 1 DMI over half of the time. This suggests that personalizing DMI recommendations may not always necessitate offering one optimal intervention, but rather offering participants a curated short list of DMIs from which they can choose or complete in combination. This may be particularly important when survivors are in periods of particularly notable emotional and physical distress, as we found evidence that adult survivors of cancer are open to multiple DMI options when they are experiencing elevated negative affect and pain and lower positive affect. This is consistent with work showing that adults use a greater number of emotion regulation strategies to cope with higher levels of distress [36], presumably due to enhanced motivation and greater support needs. Taken together, findings suggest that adult survivors of cancer may regularly be open to more than 1 DMI, especially when the need is high due to their distress.

Finally, although all interventions were endorsed throughout the study and even identified by some participants as their most preferred option, findings from this study showed that demand is not equal. Specifically, setting goals, getting support from others, and connecting with values were the least frequently endorsed interventions overall, with guided relaxation being endorsed almost 2.5 times more often than connecting with values. Because developing DMIs can be costly, researchers might consider beginning with a guided relaxation intervention before expanding their programs to values-based interventions. As researchers begin to develop specific DMIs, user-centered design practices [63] may be particularly valuable for identifying how best to bring the broad concepts of, for example, “increasing positive emotions” or “decreasing worry,” into practice through a mobile app.

### Limitations and Future Directions

Study participants self-selected to participate, largely in response to Facebook advertisements, thereby introducing a potential selection bias in our sample. Our recruitment advertising partner provided methods to target advertisements to specific demographics to increase the representiveness of our sample; however, we were limited to who ultimately responded. Moreover, our sample was predominantly composed of non-Hispanic White, female, survivors of breast cancer. Given that breast cancer in White women is the single most common type of cancer diagnosed in any demographic group, our pattern of participant demographics is not surprising. Nonetheless, it may limit the extent to which our findings generalize to non-White women and men. Moreover, we were unable to compare intervention preference differences based on sex or cancer type. However, we reran all analyses in the subsample diagnosed with breast cancer (n=232) to compare against the primary findings. These results are presented in Multimedia Appendix 1 and highlight that there is individual variability amongst survivors of breast cancer with respect to their most preferred DMIs. Although the pattern of findings between the full sample and the breast cancer subsample is generally consistent, we observed 2 primary exceptions. First, neither depression symptom severity nor past mental health diagnosis differentiated between the likelihood that a participant with breast cancer modally preferred each of the DMIs relative to guided relaxation, which suggests that current and prior depression symptoms in this subsample do not impact their general DMI preferences. Second, survivors of breast cancer currently in psychotherapy were more likely to most prefer reducing negative thoughts or setting goals relative to guided relaxation alone. Future work that increases representation will be vital in developing equitably tailored DMI systems for survivors of cancer more broadly.

We found that guided relaxation was the most preferred DMI out of the 9 interventions investigated. However, interest in guided relaxation alone was associated with better psychological functioning and better mood. Given that our sample was unselected with respect to mental health functioning and reported low levels of average momentary negative affect, interventionists targeting adult survivors of cancer with comorbid psychiatric diagnoses and worse mental health functioning might not expect the same relative DMI frequencies that we report. Relatedly, although current engagement in psychotherapy did not differentiate between overall intervention preferences in the full sample, it is possible that participants who previously engaged in psychotherapy, and found it helpful, would hold different preferences between DMIs than participants who did not find psychotherapy helpful or had no prior psychotherapy experience. Future work comparing our results to a sample endorsing higher levels of momentary negative affect and greater clinical severity would be useful. Extending this work to consider (1) intensity of interest (rather than presence or absence of interest) and (2) individual variability in intervention interest (eg, not just which intervention was most often endorsed by each participant, but the proportion of endorsement across all intervention types) would also be valuable.

The response rate for this 5-week EMA was 55%. The response rate in EMA studies is generally lower in patients with cancer than in the general population [64], given the sequelae of cancer and the added burden it incurs. Researchers should therefore be thoughtful about developing DMI-based programs for this population that require survivors to complete EMAs. Although the response rate in this study is not outside the range observed in other EMA studies [65], increasing response rates in future work will nonetheless be important to strengthen claims. For example, it is possible that participants were less likely to respond to an EMA survey when they were less interested in completing a DMI, potentially biasing our estimate of DMI interest frequency. Relatedly, participants were not explicitly given a response option to indicate no interest in an intervention. This may have created a demand effect and further inflated our estimate. However, better mood was associated with a greater likelihood to select no interest, which illustrates that participants could and did choose not to select an intervention when presumptive need was low. Nonetheless, future studies that explicitly offer participants the option to indicate no interest would be valuable. Moreover, while our question posed a hypothetical, future studies that investigate actual use of the DMIs will provide valuable insight into how interest is associated with use. We elected to start with interest rather than use to minimize resource allocation to developing less desired DMIs.

### Conclusion

Adults within 5 years of being diagnosed with cancer have heightened risk of psychological distress, and accessible mental health treatments should be developed to meet this need. Personalized and timely delivery of DMIs have strong potential to increase access to care, although researchers currently know little about adult survivors of cancer’s relative interest among intervention options and the factors impacting those preferences. In one of the largest EMA studies in adult survivors of cancer to date, we found compelling evidence that guided relaxation is most often preferred by adult survivors of cancer. Importantly, adult survivors of cancer differ in their DMI preferences between and within persons, and guided relaxation alone is typically less of interest to adult survivors of cancer when they are in greater emotional distress or have a history of mental illness. Relative spikes in pain, however, may be a particularly appropriate time to recommend a guided relaxation DMI. Taken together, results from this study advance our understanding of how to develop personalized DMI programs for adult survivors of cancer who may benefit most from their use.

## Data Availability

Deidentified data are available upon reasonable request to the corresponding author. Deidentified data are not made publicly available due to ethical considerations.

## Appendix

Multimedia Appendix 1 Results in subsample with primary breast cancer.

## Abbreviations

DMI: digital microintervention
EARS: Effortless Assessment Research System
EMA: ecological momentary assessment
GAD-7: Generalized Anxiety Disorder-7
OR: odds ratio
PHQ-8: Patient Health Questionnaire-8
REDCap: Research Electronic Data Capture
