# Supplementary material for: Identifying Time-Variant Predictors of Interest in Completing Brief Digital Mental Health Interventions Among Adult Survivors of Cancer: Ecological Momentary Assessment Study
Source: JMIR Mhealth Uhealth. 2025 Dec 18;13:e69244. doi: 10.2196/69244 (PMC12757714; doi:10.2196/69244)
Supplement: Multimedia Appendix 1 [file mhealth_v13i1e69244_app1.docx]

**Identifying Time-Variant Predictors of Interest in Completing Brief Digital Mental Health Interventions Among Adult Cancer Survivors: An Ecological Momentary Assessment Study**

APPENDIX 1

Katharine E Daniel^1^, James W. Kinchen^1,2^, Angela Chang^1^, Patrick H Finan^3^, & Philip I Chow^1^

^1^ Department of Psychiatry and Neurobehavioral Sciences, University of Virginia School of Medicine

^2^Department of Public Health Sciences, University of Virginia School of Medicine

^3^ Department of Anesthesiology, University of Virginia School of Medicine

**Funding**:

This work was supported by the University of Virginia NCI-designated Comprehensive Cancer Center

We elected to re-run all analyses reported in the main text amongst only participants with primary breast cancer to identify if any unique trends emerged in this homogenous diagnostic group. Subsample analysis results and comparisons against full sample results are presented below. See Supplemental Table 1 for demographic information for this subsample.

**Results**

13,339 randomly timed EMA surveys were submitted by 232 participants with primary breast cancer (overall response rate of 55%). No intervention was selected (*NA*) across the breast cancer subsample 1,625 times (12.18% of all surveys). All breast cancer participants selected among the interventions more often than they selected no intervention.

**Aim 1: Intervention Selection Frequency in Breast Cancer Survivors**

The most often selected intervention across this sub-sample was guided relaxation (*n* = 4,967), followed, in order, by increasing positive emotions (*n* = 4,714), reducing worry (*n* = 4,367), reducing negative thoughts (*n* = 3958), problem solving (*n* = 2403), setting goals (*n* = 2288), connecting with values (n = 1,998), getting support from others (*n* = 1,982), and other (*n* = 525). Counts sum to more than the total number of completed EMA surveys because more than one intervention could be selected at the same time. Frequency rankings between all interventions in the breast cancer sample were consistent with rankings observed in the full sample with one exception. In the breast cancer sample, connecting with values was selected more often than getting support from others.

As in the full sample, it was most common for breast cancer participants’ most preferred intervention throughout the study to be guided relaxation. Also consistent, 68.10% of breast cancer participants’ modal intervention was some other option, suggesting there is individual variability within breast cancer survivors regarding each participant’s most preferred intervention.

**Aim Two: Person-Level Predictors of Overall Intervention Preference in Breast Cancer Survivors**

A series of multinomial logistic regressions were conducted to examine the predictive effect of each predictor on the log odds that a participant preferred each of the interventions relative to guided relaxation following steps outlined in the main text.

**Age.** Specifying age provided unique predictive information relative to the null model (X^2^(9) = 26.40, *p* < .01). As observed in the full sample, results showed that as participant age increased, participants became increasingly likely to prefer problem solving relative to guided relaxation (b = .247, SE = .09, Wald Statistic = 2.88, 95% CI = .08 - .42, OR = 1.28). Unlike in the full sample, age did not predict relative interest between guided relaxation and some other intervention. As in the full sample, no other coefficients were statistically significant.

**Currently in Active Cancer Treatment.** As observed in the full sample, specifying cancer treatment status provided no unique predictive information relative to the null model (X^2^(9) = 9.99, *p* = .351).

**Currently in Active Psychotherapy.** Unlike in the full sample, specifying psychotherapy treatment status provided unique predictive information relative to the null model (X^2^(9) = 23.71, *p* < .01). Among breast cancer survivors, participants currently in (vs. not in) psychotherapy were more likely to prefer reducing negative thoughts (b = 1.98, SE = .65, Wald Statistic = 3.05, 95% CI = .71 – 3.25, OR = 7.23) and setting goals (b = 1.98, SE = .88, Wald Statistic = 2.25, 95% CI = .26 – 3.70, OR = 7.23) relative to guided relaxation alone. No other coefficients were statistically significant.

**History of Major Depressive Disorder.** Unlike in the full sample, specifying depression diagnostic history provided no unique predictive information relative to the null model (X^2^(9) = 16.77, *p* = .052).

**Current Depression Symptom Severity.** Unlike in the full sample, specifying depression symptom severity provided no unique predictive information relative to the null model (X^2^(9) = 6.13, *p* = .727).

**History of Anxiety Disorder.** As observed in the full sample, specifying anxiety diagnostic history provided no unique predictive information relative to the null model (X^2^(9) = 5.83, *p* = .757).

**Current Generalized Anxiety Symptom Severity.** As observed in the full sample, specifying generalized anxiety symptom severity provided unique predictive information relative to the null model (X^2^(9) = 18.9, *p* < .05). Like in the full sample, as generalized anxiety symptom severity increased, breast cancer participants became increasingly likely to prefer reducing worry (b = .14 , SE = .05, Wald Statistic = 3,08, 95% CI = .05 - .23, OR = 1.15), reducing negative thoughts (b = .13, SE = .06, Wald Statistic = 2.06, 95% CI = .01 - .25, OR = 1.14), and getting support from others (b = .22, SE = .07, Wald Statistic = 2.95, 95% CI = .07 - .36, OR = 1.24) relative to guided relaxation alone. Unlike in the full sample, anxiety symptom severity did not predict relative interest between guided relaxation alone and increasing positive emotions nor preferring multiple brief interventions. As in the full sample, no other coefficients were statistically significant.

**Aim 3: Situation-Level Predictors of Momentary Intervention Interest Amongst Breast Cancer Survivors**

**Aim 3a. Likelihood to express interest in any intervention versus no interest.**  A series of multilevel logistic regressions were conducted to examine the predictive effect of between- and within-person negative affect, positive affect, or pain on the log odds that breast cancer participants endorsed no (vs. any) interest in an intervention following steps outlined in the main text.

***Negative Affect.*** As observed in the full sample, higher levels of within-person negative affect was associated with greater likelihood of reporting interest in an intervention (b = -.08, SE = .01, p < .001, OR = .92). Unlike in the full sample, between-person negative affect was not a significant predictor (b = -.13, SE = .07, p = .072, OR = .88).

***Positive Affect.*** As observed in the full sample, higher level of within-person positive affect was associated with a lower likelihood of reporting interest in an intervention (b = .05, SE = .01, p < .001, OR = 1.05). Also consistent with the full sample, between-person positive affect was not significant (b = -.002, SE = .05, p = .777, OR =1.02).

***Pain****.* As observed in the full sample, higher levels of between-person pain was associated with greater likelihood of reporting interest in an intervention (b = -.40, SE = .16, p < .05, OR = .67). Unlike in the full sample, within-person pain was not a significant predictor (b = -.05, SE = .03, p = .174, OR = .95).

**Aim 3b. Likelihood to express interest in any single intervention versus multiple interventions.** A series of multilevel logistic regression was conducted to examine the predictive effect of between- and within-person negative affect, positive affect, or pain on the log odds that breast cancer participants endorsed interest in multiple (vs. a single) interventions following steps outlined in the main text.

***Negative Affect*.** As observed in the full sample, higher levels of within- (b = .08, SE = .01, p < .001, OR = 1.08) and between-person negative affect (b = .20, SE = .04, p < .001, OR = 1.22) were associated with a greater likelihood of reporting interest in multiple interventions.

***Positive Affect.*** As observed in the full sample, higher levels of within- (b = -.04, SE = .01, p < .001, OR = .96) and between-person positive affect (b = -.09, SE = .03, p < .01, OR = .91) were associated with a lower likelihood of reporting interest in multiple interventions.

***Pain****.* As observed in the full sample, higher levels of within- (b = .07, SE = .02, p < .01, OR = 1.08) and between-person pain (b = .38, SE = .10, p < .001, OR = 1.46) were associated with a greater likelihood of reporting interest in multiple interventions.

**Aim 3c. Likelihood to express relative interest among specific interventions.** A series of multilevel multinomial logistic regressions were conducted to examine the predictive effect of within- and between-person negative affect, positive affect, and pain on the log odds that breast cancer participants selected each of the interventions relative to guided relaxation following steps outlined in the main text.

***Negative Affect*.** As observed in the full sample, higher levels of within- and between-person negative affect were associated with a significantly greater likelihood of reporting interest in each of the following interventions relative to guided relaxation: increasing positive emotions (within: b = .05, SE = .02, *p* < .01; between: b = .12, SE = .05, *p* < .05), reducing worry (within: b = .12, SE = .02, *p* < .001; between: b = .22 , SE = .05, *p* < .001), reducing negative thoughts (within: b = .15, SE = .02, *p* < .001 ; between: b = .20, SE = .05, *p* < .001), problem solving (within: b = .13, SE = .02, *p* < .001; between: b = .13, SE = .05, *p* < .01), getting support from others (within: b = .18, SE = .02, *p* < .001; between: b = .19, SE = .04, *p* < .001), and other (within: b = .13, SE = .02, *p* < .001; between: b = .11, SE = .05, *p* < .05). Unlike what was observed in the full sample, within- and between-person negative affect did not significantly differentiate between likelihood of selecting connecting with values or setting goals relative to guided relaxation.

***Positive Affect*.** As observed in the full sample, higher levels of within- and between-person positive affect were associated with a significantly lower likelihood of reporting interest in each of the following interventions relative to guided relaxation: increasing positive emotions (within: b =-.04, SE = .01, *p* < .001; between: b = -.06, SE = .03, *p* < .05), reducing worry (within: b =-.05, SE = .01, *p* < .001; between: b = -.07, SE = .03, *p* < .01), reducing negative thoughts (within: b = -.08, SE = .01, *p* < .001; between: b = -.06, SE = .03, *p* < .05), getting support from others (within: b = -.07, SE = .02, *p* < .001; between: b = -.10, SE = .03, *p* < .001). Also consistent with the full sample, higher levels of within-person positive affect (but not between-person positive affect) were associated with a significantly greater likelihood of reporting interest in setting goals (b = .05, SE = .01, *p* < .001) and connecting with values (b = .04, SE = .02, *p* < .05) relative to guided relaxation. Unlike what was observed in the full sample, neither within- nor between-person positive affect differentiated between likelihood to select another intervention relative to guided relaxation and only within-person positive affect significantly differentiated between likelihood to select problem solving relative to guided relaxation (b = -.06, SE = .01, *p* < .001).

***Pain.*** As observed in the full sample, higher levels of within-person pain (but not between-person pain) were associated with lower likelihood of reporting interest in setting goals (b = -.18, SE = .06, *p* < .01) and connecting with values (b = -.16, SE = .07, *p* < .05) relative to guided relaxation. Also consistent with the full sample, higher levels of between-person pain (but not within-person pain) were associated with greater likelihood of reporting interest in reducing negative thoughts (b = .19, SE = .09, *p* < .05), reducing worry (b = .36, SE = .13, *p* < .001), and getting support from others (b = .26, SE = .09, *p* < .01) relative to guided relaxation. Unlike in the full sample, neither within- nor between-person pain differentiated between likelihood to select another intervention or problem solving relative to guided relaxation. Moreover, only within-person pain significantly predicted likelihood to select increasing positive emotions relative to guided relaxation (b = -13, SE = .05, p < .01).

**Supplemental Tables**

**Table S1.** *Breast Cancer Sample Demographics*

| **Participant Characteristics** | ***n* (%) or *M* (*SD*)** |
| --- | --- |
| Age | 47.98 (*SD* = 10.52) |
| Sex |  |
| Male | 0 (0%) |
| Female | 231 (99.57%) |
| Some Other Sex | 1 (.43%) |
| Race |  |
| White | 202 (87.45%) |
| Black | 9 (3.90%) |
| American Indian/Alaska Native | 4 (1.73%) |
| Asian | 12 (5.19%) |
| Native Hawaiian/Pacific Islander | 0 (0%) |
| Some Other Race | 4 (1.73%) |
| Ethnicity |  |
| Hispanic | 210 (92.11%) |
| Non-Hispanic | 18 (7.89%) |
| **Cancer Information** |  |
| In Active Cancer Treatment | 48 (21.52%) |
| **Psychiatric Information** |  |
| In Active Therapy | 75 (32.47%) |
| History of Major Depressive Disorder | 110 (47.41%) |
| History of Anxiety Disorder | 119 (51.29%) |
| PHQ-8 | 7.72 (SD = 5.09) |
| GAD-7 | 5.57 (SD = 4.76) |

PHQ-8 = measure of depression symptoms. GAD-7 = measure of generalized anxiety symptoms.
